# Analyzing the sources and nature of influence: how the Avahan program used evidence to influence HIV/AIDS prevention policy in India

**DOI:** 10.1186/1748-5908-8-44

**Published:** 2013-04-17

**Authors:** Nhan T Tran, Sara C Bennett, Rituparna Bishnu, Suneeta Singh

**Affiliations:** 1 Alliance for Health Policy and Systems Research, World Health Organization, Geneva, Switzerland; 2 Health Systems Program, Department of International Health, Johns Hopkins Bloomberg School of Public Health, Baltimore, Maryland, USA; 3 Tolly Niket, N.S.C. Bose Rd, Kolkata, India; 4 Amaltas, Hauz Khas, New Delhi, India

**Keywords:** Evidence use, HIV/AIDS, Policy influence

## Abstract

**Background:**

Major investments by development partners in low- and middle-income countries (LMICs) often seek to develop a supportive policy environment. There is limited knowledge about the mechanisms that development partners use to influence government policy, or which mechanisms are effective. This study assessed the influence of Avahan, a large HIV/AIDS prevention program in India supported by the Bill and Melinda Gates Foundation, on the development of HIV/AIDS policies in India, particularly the National AIDS Control Program III (NACP III).

**Methods:**

A retrospective assessment of the contributions of Avahan to the development of NACP III was conducted based upon document review and in-depth interviews with key informants, including Avahan staff and staff of implementing partners. This assessment was carried out within a framework centered on three domains: evidence considered by policy and decision-makers; the channel through which influence is exerted; and the target audience for influence.

**Results:**

Respondents identified a number of respects in which Avahan influenced NACP III policy, notably, Avahan influenced perception of the feasibility of scaling up services (through a demonstration effect) and Avahan, along with others, helped ensure a strong focus on targeted interventions. Overall Avahan’s influence was greatest during policy implementation. While the extent to which research evidence generated by Avahan influenced NACP III was limited, best practice evidence generated by Avahan, including the lessons learned from routine implementation and management, contributed significantly to NACP III. This was largely due to the credibility Avahan had established and strategic ‘inside track’ communications.

**Conclusion:**

While studies of knowledge translation typically focus primarily on scientific evidence, this study suggests that other forms of evidence, notably best practice evidence derived from program experience, and disseminated through personal communication, were particularly influential. The framework developed for the paper provides a useful tool to analyze how evidence-based influence is exerted.

## Background

In many low- and middle-income countries (LMICs) development partners are often thought to be highly influential in terms of shaping government policies and priorities. Analysts have suggested that the financial clout of such development partners is the primary source of their influence [1,2]. However it has also been shown that aid conditionality (whereby the disbursement of aid is contingent upon specific policy reforms) is unlikely to be effective without buy-in from the national government [3,4]. Donor influence may be based upon different sources of power, including access to information and expertise, and many development partners seek to employ evidence and expertise to inform and shape policy debates. Indeed, Morrissey argues that a key role for aid is to inform and support policy processes [3].

There is relatively limited academic or empirical work that explores the processes through which development partners use knowledge to influence national government policy, and much of the available literature focuses on how research evidence may influence policy [5-7]. However there are multiple other forms of evidence - tacit or experiential knowledge, best practice knowledge, norms, and guidelines - that may also influence policy and decision making. This study sought to understand how one particular development program, the Avahan program, supported by the Bill and Melinda Gates Foundation (BMGF), used evidence, in its various forms, to influence national aids control policy in India.

**Figure 1:**
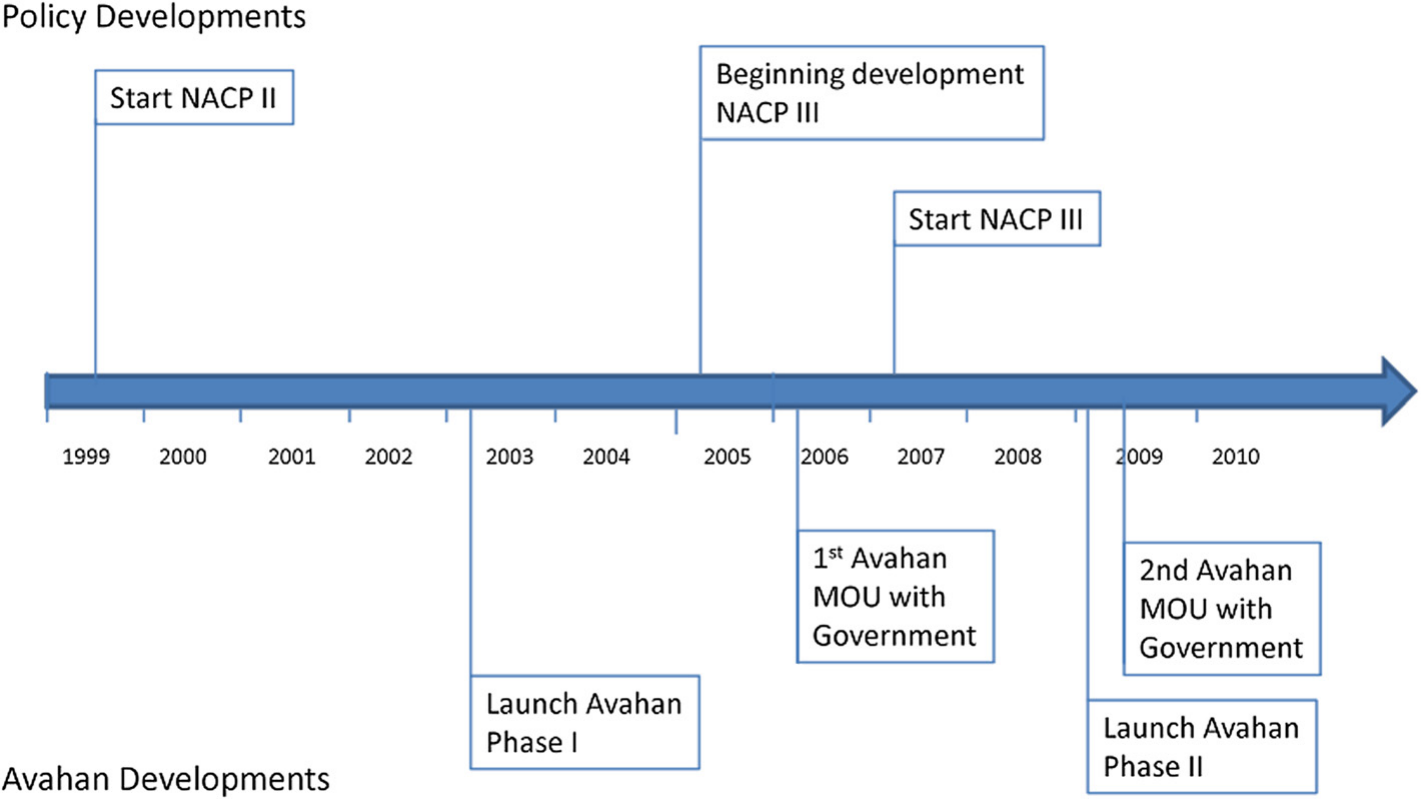
Timeline for development of Avahan and NACP III.

Initiated in 2003 (see Figure 1), the Avahan program represents a major investment by BMGF to address the spread of HIV/AIDS in India. While supporting service provision in the six high-prevalence states at the time (four southern states: Andhra Pradesh, Tamil Nadu, Maharashtra, and Karnataka and two north eastern states: Manipur and Nagaland), the Avahan program has also sought to influence national policy. In particular, the program has sought to maintain a focus on most at-risk populations, and the scale-up of effective HIV/AIDS prevention services to address the needs of these populations. During the life of the Avahan program, the main HIV/AIDS policy framework in India has been the National AIDS Control Program III (NACP III) (NACO 2006). This 250-page policy document, developed during 2005, provides a comprehensive description of national AIDS control policies in India. Our analysis focuses in particular on the influence of Avahan on the development and implementation of NACP III.

### Conceptual framework

**Figure 2:**
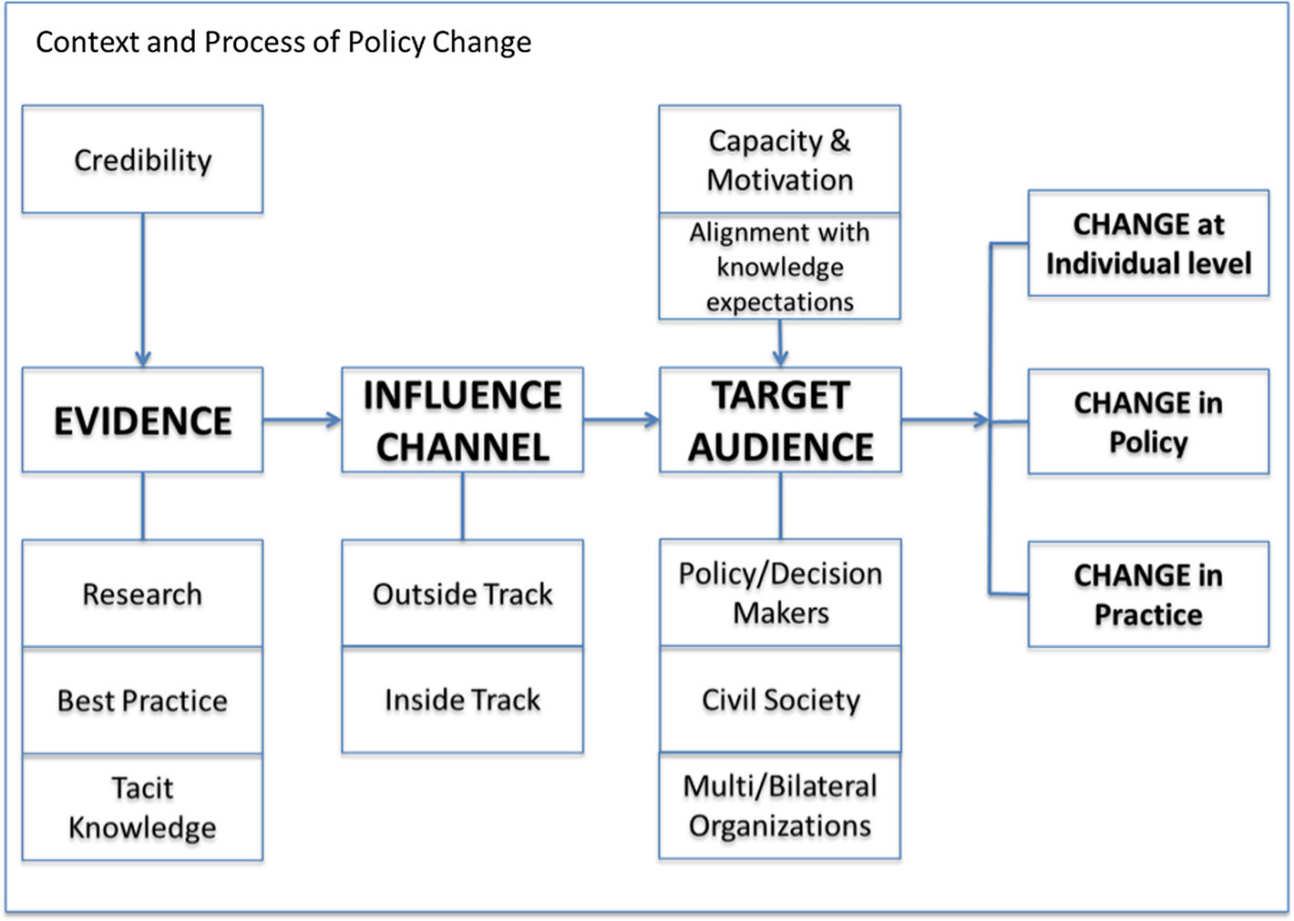
Conceptual framework.

We developed a conceptual framework that draws upon previous publications in this field [8,9] to describe how the Avahan program may have influenced the development of NACP III. This framework (Figure 2) is centered on three domains: evidence considered by policy and decision-makers; the channel through which influence is exerted; and the target audience for influence. The framework identifies three levels of change where influence can be observed:

1. Changes at the individual level, such as shifts in attitudes, which may not always be reflected in policy or practice, but are nonetheless an important indicator of influence and may be a precursor to other types of change.

2. Changes in policy, such as formal rules and regulations governing programs at the national, state, or local levels.

3. Changes in practice, which are needed for policies to have an impact and can relate to activities ranging from clinical to management.

All of these changes occur within a specific social, political, and economic environment.

Our framework considers three categories of evidence, acknowledging that there may be some overlap and interaction between these different categories:

1. Research evidence including results from dedicated studies that seek to address specific questions, analysis of epidemiological or survey data, modeling, or syntheses of existing evidence. This type of evidence is generally disseminated through peer-reviewed publications and scientific meetings.

2. Best practice evidence comes from practice and represents practical learning from the field. This includes learning and data that are gathered through routine program management activities. Although ‘best practices’ are sometimes presented in the peer-reviewed literature, they do not represent findings from dedicated research activities and are more often published in the form of monographs/reports, and shared through conferences, meetings, and site visits along with other forms of personal communication.

3. Tacit knowledge represents learning generated from personal experiences [10]. It may often be introduced into the decision-making process by civil society organizations to ensure that the experiences of vulnerable populations are considered, but also includes the tacit knowledge of program managers and other implementers.

The target audience for influence includes policy makers, civil society, and multi- and bi-lateral organizations. While government policy makers take primary responsibility for policy formulation and implementation, the other actors may influence policy [11,12]. The uptake of evidence and its consideration in the decision-making process is further influenced by the credibility of the source of evidence and the channel through which it is communicated. The framework also considers factors that impact the use of evidence, including both the motivation and capacity of the policy- or decision-maker, and the extent to which evidence is aligned with their prior beliefs and knowledge.

Inside track communication is based on personal or professional relationships that operate from within the government decision-making system, such as through direct engagement with decision-making bodies or through active representation on governing councils and boards. Such an approach allows the organization seeking to influence policy, to better understand the information needs of decision-makers, to tailor messages accordingly and ensure that they reach policy makers in a timely manner. The ‘outside track’ refers to communication that is generated outside of the government decision-making system and is perceived as external to the system. While external communication can sometimes result in a perception of greater objectivity and lend credibility to the messages because the messages are not subject to internal pressures and demands of the government decision-making system, it may also limit opportunities for the ideas to be considered during internal government discussions.

## Methods

The time period addressed by this analysis begins in 2005 and runs through to 2009, focusing on the period during which NACP III was formulated and early years of implementation.

The analysis is retrospective, conducted through document review and semi-structured interviews that were conducted between October 2010 and March 2011. Documents reviewed included NACP II and NACP III policy documents, and guidelines and operating procedures related to NACP III, Avahan communications including monographs on best practice (BMGF 2008, 2009, and 2010), the Avahan Common Minimum Program, and Avahan research-oriented publications. Actors involved in the development of NACP III were identified as belonging to four main groups, namely: policy- and decision-makers from NACO and State AIDS Control Society (SACS); staff of multi- and bilateral aid agencies and international organizations; civil society representatives, particularly local academics and staff of NGOs who were involved with the development of NACP III; and foundation staff in India, and the staff of Avahan implementing partners. Semi-structured interviews were carried out with representatives from each of these groups during the period October 2010 and March 2011, with 27 interviews conducted in total, relatively evenly spread across each group.

Interview guides asked respondents to reflect on the shifts between NACP II (1999 to 2006) and NACP III (2006 to 2011) in both policy and implementation, probed their understanding of why these shifts took place, and in particular what role Avahan may have played in the shifts, and then asked respondents to consider the nature of communications from Avahan and the channels through which influence was exercised.

Interviews were recorded and transcribed. Coding was conducted using a framework approach, that is thought to be particularly appropriate for health policy studies [13]. The constructs in the conceptual framework were used to identify an initial set of codes that were then adapted and added to based on the content of the transcripts. In analyzing findings we sought to triangulate between four different stakeholder groups (government, development partners, civil society, and foundation staff and Avahan grantees, collectively referred to as ‘Avahan’), to assess the extent to which there were similar perspectives on the extent and nature of influence exercised by Avahan. Findings reported below represent perspectives that were shared across at least two stakeholder groups, unless otherwise noted.

The research protocol and instruments were submitted to Johns Hopkins Bloomberg School of Public Health Institutional Review Board which determined that this was not human subjects research. Within India the YRG Care Institutional Review Board reviewed and approved the study.

## Results

### Context and process

NACP III was developed at a point where there was recognition that HIV/AIDS policy needed to change: this perspective was driven by learning from NACP I and NACP II, and was supported by a growing political commitment to address HIV/AIDS in India. During the mid 2000s, around the time NACP III was being developed, there was substantial debate about the scale of the epidemic in India [14-17] with some analysts asserting that India was ‘on the brink of a significant epidemic’ [17]. Debates took place about whether the required response should be that typically associated with a generalized or concentrated epidemic [18,19].

NACO played a strong leadership role in the development of NACP III and was wedded to an open, participatory, and evidence-informed policy formulation process. Planning started in early 2005 with a retreat to reflect on lessons learned from NACP II, following which NACO established a national planning team and developed a framework for the new policy [20]. Fourteen thematic working groups were established to address different program areas (such as condom programming, financial management etc.). Program planning was conducted through an extremely consultative process that included an online consultation as well as face-to-face consultations at state and national levels. A number of dialogues with development partners (such as the World Bank, USAID, *et al*.) were also hosted. These extensive consultations were perceived to lend greater credibility to the final outcome:

‘…I mean talking about leadership, a lot of mobilization at the political level had been done through members of parliament—there was a parliamentary group of MPs who were also active. So, all that really lent support to the entire process and one felt confident going about one’s task.’ (NACO #3)

‘See, NACP I was basically administrative response, because the country was not ready to face up to the challenge…these great initiatives were taken during NACP II. Now, NACP III, when this planning started, we wanted to build on that.’ (NACO #1)

‘I think everybody appreciated that it was such a transparent and open process. I have never heard of any national program to be so inclusive in the development process, so inclusive and so open to feedback and inputs, I have never seen that and I don’t think it will ever happen like that again.’ (Civil Society #2)

### The nature and credibility of evidence

Although Avahan always intended to support research, it did not have an explicit research agenda at the inception of the program (it took time for the evaluation agenda to be formulated and evaluation grants were only awarded in June 2004). Further, at the time when planning for NACP III started, Avahan had less than two years of operational experience. Avahan and its partners generated lessons that were disseminated in the peer-reviewed and grey literature but there were no substantive publications at the time of NACP III planning, and Avahan evidence did not appear to be a primary source of evidence considered during the development process of NACP III. When questioned about Avahan studies that were considered during the NACP III development process, none of the respondents interviewed recalled any specific study by Avahan that was explicitly considered as evidence in the development of NACP III. This may in part be due to the fact that draft reports and data were already being shared with NACO and other stakeholders prior to publication in journals:

‘I do think that the international publications have been useful not so much internally but I think globally those publications have been important. The peer review journal publications have been important.’ (Avahan #6)

‘…Publications, no not really except their study [indiscernible] which, of course, took a long time and that came out with some evidence. But their presentations were… all along throughout the period they would come up with some presentation…they did give the data to say what was being done and so on…’ (NACO #6)

While, the extent to which the research evidence generated by Avahan influenced NACP III was limited, best practice evidence generated by Avahan, including the lessons learned from routine implementation and management, contributed significantly to the development process for NACP III. Specifically, the management of the program with rigorous monitoring, the focus on high-risk target populations—particularly the use of differential strategies for each population, and use of norms and standards were incorporated in NACP III. These ‘best practices’ and lessons learned clearly resonated with the Government of India (GOI) and NACO, and had a significant impact on the overall development of NACP III:

‘…the best way to make the government agencies to look at you and say oh, these are the experience of this agency,…It is primarily making a sharing exercises. It is something like a workshop like or a seminar like activity in which the senior level top officials of the government are invited and they do some sort of a sharing of the experiences…all those things contributed significantly in the formulation of the NACP III.’ (NACO #2)

In addition to disseminating its best practices, Avahan also facilitated many field visits that allowed NACO and other stakeholders to see firsthand how the programs were operating. A senior member of the NACO staff recalled the impact of visiting an Avahan site and seeing the work being carried out and the impression that it made. This ‘tacit evidence’ appeared to be influential in policy formulation and especially during the implementation of NACP III:

‘My visit to the Gates funded program in Mysore, and there I saw for myself…So, I did think at that time that if this was possible in such a short period of time through program inputs in a focused manner being provided, then it should be possible to scale it up throughout the country too. So, the concept of scaling up really came with my visit there.’ (NACO #6)

Avahan’s credibility was an important factor in facilitating the influence that it was able to achieve. This credibility was earned and not accorded to Avahan simply because it was a product of BMGF. In fact, as a relative newcomer to the HIV/AIDS scene, Avahan was initially met with a certain degree of skepticism that needed to be overcome. However, through the successful implementation and scale up of its targeted intervention programs, Avahan gained credibility within the eyes of NACO and other stakeholders. In particular respondents noted the business acumen that Avahan brought to HIV/AIDS prevention and scale-up, and recognized the management expertise it offered. Avahan credibility therefore was due to its ability to generate and document positive results following the implementation of activities. This perception was shared among other stakeholders in different states and was a facilitating factor for Avahan to be able to influence programs across India. It also served to reinforce the value of the program to the government. As indicated by several respondents:

‘That Avahan was successful…Otherwise people in other states would not have asked us to ask Avahan to extend their program to their states. So, that was an indicator that the Avahan was popular.’ (NACO #1)

‘So there were many who try to influence them but didn’t manage and then there were some who managed because it resonated.’ (Int'l Organization #3)

The credibility gained from early successes facilitated the uptake of information by target audiences. This appears to have created a virtuous cycle: as the government began to accept the work of Avahan, this lent further credibility to Avahan and its learning.

### Influence channels

Avahan and its partners employed a number of channels to communicate lessons learned to the Government of India and other stakeholders, from hosting site visits to participating in planning meetings, presenting data to publication of in-country reports, newsletters, and from 2008 onwards monographs, and to a lesser extent peer-reviewed literature. As summarized in Table 1, some of these approaches allowed Avahan to exercise influence within government decision-making processes (inside track), while others communicated lessons to a broader audience, external to government (outside track). The types of evidence communicated varied among the communication channels. Research evidence was largely used to communicate to external organizations, while best practices and tacit evidence were shared more frequently through 'inside' channels of communication. The majority of Avahan communication activities were carried out during implementation phases of NACP III when Avahan had more knowledge on best practices that could be scaled up.

**Table 1:** Avahan influence strategies for the development of NACP-III

**Strategy**	**Type of evidence**	**Communication channel**	**Target audience**	**Timing of communication**
Site-visits			Govt (NACO)	
	Tacit Knowledge	Outside Track	Multilateral Org	During, Post-NACP III Development
	Best Practice		Civil Society	
			International	
Presentations	Best Practice		Govt (NACO)	
	Research	Outside Track	Multilateral Org	During, Post-NACP III Development
			Civil Society	
Personal contact with NACO	Best Practice			During, Post-NACP III Development
	Research	Inside Track	Govt (NACO)	
	Tacit Knowledge			
Participation on NACP III Workgroups			Govt (NACO)	
	Best Practice	Inside Track	Multilateral Org	During NACP III Development
	Research		Civil Society	
Guidelines	Best Practice	Inside Track	Govt (NACO)	Post-NACP III Development
	Research		Civil Society	
Reports/Monographs			Govt (NACO)	
	Best Practice	Outside Track	Multilateral Org	During, Post-NACP III Development
			Civil Society	
			International	
Staffing support for NACO	Best Practice	Inside Track	Govt (NACO)	Post-NACP III Development
	Tacit Knowledge			
Management of TSUs	Best Practice	Inside Track	Govt (SACS)	Post-NACP III Development
	Tacit Knowledge			
Peer-reviewed literature	Research	Outside Track	Govt (NACO)	Post-NACP III Development
			Multilateral Org	
			International	

Avahan’s effectiveness in influencing policy implementation and practice was achieved primarily by inside-track communication where it engaged with NACO and SACS on a more personal level and in many respects integrated itself into NACO. Avahan supported advisory staff positions within NACO that enabled it to access internal discussions and dialogues at which few other external agencies were present. Positioned within NACO, foundation and Avahan staff were able to engage in informal communications, participate in executive meetings, and other working group discussions where data were presented and informal exchanges of ideas took place. This close relationship with the government was a key factor in Avahan’s influence on NACP III and was something that even those outside of NACO and Avahan observed:

‘…the fact that Avahan participated directly in supporting NACO and putting staff inside NACO to help with the implementation of NACP III …….have made things much easier. So the perception was definitely better this time.’ (Int'l Organization #1)

‘…by embedding themselves within the national program in the form of trying to help in putting together the Technical Support Unit was, I think, something that contributed to the advancement of the implementation.…’ (Civil Society #7)

‘You just cannot say, ‘Okay let me teach you what it is and go in for two hours.’ It does not work. So, that is what does not work. And, therefore, what works – if you sit with people for a year – and that’s what I mean, ‘sit with people;’ not sit saying, ‘I will come for one day today and one day after one month.’ So, the only other way is to sit for at least two to three years full time with people – which is what we did…. The same logic holds true during the SACS where you would work with them to place multiple people in the SACS who worked in Avahan and, so, there is transfer of knowledge by, actually, working together as opposed to only presenting presentations. So, I would say that by far, that is the biggest…’ (Avahan #2)

Being positioned within NACO also helped to generate greater NACO ownership of lessons derived from Avahan. There was consensus from all stakeholder groups that these ‘inside track’ approaches to communication, greatly contributed to Avahan’s credibility and influence.

Although Avahan focused more on ‘inside track’ communication channels, it did to a lesser extent employ ‘outside track’ channels of communication. This communication served largely to increase visibility and to stimulate discussions of the Avahan model internationally more than to communicate with the Government of India and other stakeholders in India. These types of communication were also important for shaping Avahan’s recognition and credibility globally. Overall, Avahan appeared to neglect communications with stakeholders in India outside of government and did not apply the same ‘inside track’ strategies to their engagement with other donors and international organizations, this occasionally led to mistrust on the part of such actors.

### Impacts of Avahan influence

#### Changes in attitudes

Respondents identified a number of substantial shifts in attitude between NACP II and NACP III, noting in particular shifting perspectives towards working at scale, the role of the community, and the use of data and evidence. The issue of scale was the area where the most dramatic shift in perspective occurred and this was widely seen by respondents. While there was a shift in attitude towards the necessary scale for implementing targeted interventions in particular, the government was also perceived to be intent on scaling up many other services, such as treatment services. The second area where a substantive shift in attitude occurred was around community mobilization, with a much more prominent role given to communities. Finally, with respect to the use of evidence, respondents observed an intensified effort during NACP III to use evidence for program monitoring and planning.

Interviews gave a nuanced picture of Avahan’s contribution to changes in attitudes, which is reinforced by the broader picture (described above) of the context in which NACP III was developed. Concerning scale up, respondents within NACO thought that the Avahan experience did influence perception of the feasibility of scaling up, primarily through a demonstration effect. In terms of a shift towards evidence-based planning, some respondents including those from NACO attributed an important role to Avahan, but there were clearly multiple other influential factors. One representative of an international organization noted that this issue was one where ‘a lot of partners also were helping the Government to get to that point’ (International Organization #5), and the stronger emphasis upon evidence use in planning could be seen as a natural evolution with a gradually growing sophistication in planning and policy approaches.

#### Changes in policy

The policy shifts that took place built upon the attitudinal shifts described above. First, while HIV/AIDS prevention was as much a focus of NACP II as NACP III, during NACP II much of the preventive focus of targeted interventions was on ‘composite groups [14]. Thus, a single targeted intervention frequently addressed multiple different types of most at-risk populations [21]. NACP III focused targeted interventions on a particular most at-risk population and indeed a whole section in NACP III covers ‘differential strategies’ that distinguish between different populations. Most groups of actors interviewed acknowledged a role for Avahan in terms of ensuring a clear emphasis upon targeted interventions for target populations:

‘Avahan has shown how do you choose the most strategic population, more strategic intervention, put their money in and get the key results, the most effective results’ (Civil Society #4).

However, the focus on targeted interventions was supported by emerging evidence, and could also be viewed as a natural progression in the development of the program.

Of the different aspects of policy change between NACP II and NACP III, there are a few additional areas where Avahan’s contribution stands out, such as the focus on community-based organizations. NACP III also reflected a stronger focus on strengthening capacities, and monitoring and evaluation (M&E) systems. While the Avahan program certainly supported a strong focus on M&E and capacity development, it appears that Avahan’s contribution to these elements was less at the policy development stage, and more in terms of providing practical support to implementing NACO’s vision (see changes in practice below). Other important aspects of the policy shift between NACP II and NACP III, such as the focus on decentralization and integration with existing services, were not part of Avahan’s influence agenda and appear to have been brought about by other forces, particularly those internal to government.

#### Changes in practice

Avahan’s contribution was particularly great in terms of changes in practice. Respondents identified a number of areas where practice changed during the implementation of NACP III. One notable area where Avahan appeared to have had a significant impact was around improvements in the M&E systems with tracking systems for different high-risk groups and stronger data management established throughout the system (NACO #2, 4, 5, 7 and International organization #2). Several respondents traced this effect back to Avahan. One specific example given concerned the critical link between pay and performance. While it is a hallmark of Avahan that NGOs are tightly held to performance criteria it is typically rare for governments to cancel contracts due to non-performance, but one respondent cited the fact that under NACP III, NACO had cancelled a lot of contracts with underperforming NGOs as evidence of Avahan influence in this field.

In terms of the management of the program, there was a general sense from respondents that the implementation of NACP III was ‘much more professional’ (Civil society #2) and ‘highly structured’ (Civil society #1) with a stronger focus on getting ‘better results at district level’ (International Organization #4). Respondents from all the different respondent groups articulated an important role for Avahan in terms of influencing the overall management approach taken to NACP III:

‘Early days those were the things that we religiously got out under [name] leadership but we got all of these documents written up and documented various critical aspects of our program. So at least it was on paper that this is how it works within Avahan whether it’s crisis management, whether it’s IDU programming, whether it’s MSM programming etcetera. So stuff has been documented in terms of monographs.’ (Avahan #1)

‘I think Avahan has enormous capacity to carry things forward to its endpoint particularly in planning. And therefore I would rate it as – the states are actually embracing the Avahan inputs quite significantly.’ (Civil Society #7)

‘It comes out from a strong management system that they produced. I think that model is what NACP III can learn from.’ (Civil Society #4)

Specific management examples given included the clear supervisory structure which was developed under NACP III with every ten targeted interventions (TIs) having one supervisor assigned to oversee them (NACO #6, Civil Society #1). The presence of one supervisor for every ten targeted interventions was also seen to be a very direct influence of Avahan, building directly on Avahan’s own standards. Further, standards were developed that required the supervisor to make regular (monthly) visits to each TI:

‘BMGF …..sort of convey this requirement that program management at NACO level for the TIs is also a great requirement and that is how they sort of asked them to increase—or what do you say—strengthen the program management at TI, of TIs at NACO level.’ (NACO #4)

Finally, Avahan played a critical role in the development of the quasi-governmental technical units (known as technical support units) that advised NACO and SACS. While these entities existed prior to NACP III they were considerably strengthened during NACP III, and Avahan played a significant role in this. Technical support units (TSU) were established at the National level (NTSU) with specific technical support groups (TSGs) for the condom and trucker programs. These units provided support on priority setting, management, and funding. At the state levels, TSUs provided technical and managerial support for program implementation. Staff from Avahan and other international organizations cited these units as well as the development of operational guidance to help facilitate implementation of best practice models, as being a substantive component of their work in India:

‘We worked with them for two years to write the operational guidelines which were all based on Avahan learnings, including the community mobilisation aspects.... And without exaggeration, there must be some 50 tools which were used, and they were all given to NACO in NACP III.’ (Avahan #2)

‘So, all those guidelines were being prepared. What I saw was the products which were progressively getting much better and I was told that the technical members of the Avahan people, they participate in these guidelines.’ (Int’l Organization #5)

‘Half our effort with NACO is really on the institutional mechanisms, which have been the Technical Support Units and the National Technical Support Unit and the Technical Support Groups and how to set it up, and what is their role and defining all that.’ (Avahan #2)

Avahan championed such structures, and the management style that Avahan advocated for—a strong focus on results, close monitoring and supervision—required substantial technical oversight. Perhaps for these reasons, several respondents saw these quasi-governmental agencies as being very much an Avahan creation, although the first such technical support units were actually created with support from the United Kingdom Department for International Development and were known as program support units. Further, Avahan directly supported the National Technical Support Unit, the Technical Support Units in Karnataka and Tamil Nadu, and both the condom and truckers Technical Support Groups.

## Discussion

There were undoubtedly many factors that influenced the development of NACP III. Avahan was one among several factors that contributed to the policy development process and had some success in influencing NACP III. Much of its influence was exercised during the implementation period, and focused on the management and operational procedures necessary for implementing prevention activities, particularly those involving most at risk populations. Avahan’s influence during the period of formulating NACP III was less marked. While Avahan staff participated actively on working groups that were responsible for formulating NACP III this process occurred very early during the life of Avahan. Certainly, shifts between NACP II and NACP III reflect some of the critical points that Avahan was seeking to promote, but informants did not describe a major role for Avahan at this juncture. It should be noted that it was not until very recently (during the period that this research was conducted) that Avahan developed an explicit strategy for its influence.

Avahan’s influence was based in good part on evidence, however the most influential forms of evidence appear to have been best practice evidence, notably operational norms and guidelines, and to some extent tacit evidence derived from field visits by government staff to Avahan service delivery sites. What is typically thought of as ‘evidence,’ particularly research findings, or scientific data from surveillance systems, appears to have had less impact. However, this finding needs to be interpreted with caution. First, and for the reasons of timing noted above, Avahan was more influential during policy implementation than policy formulation. Questions concerning implementation require studies of processes that were not the focus of Avahan’s investment in research, which primarily explored trends in the epidemic, and conducting impact evaluations of interventions. While the findings from impact evaluations are likely to be relevant to questions regarding the effectiveness of the Avahan model, these types of studies do not answer the ‘how to’ questions that are critical to the replication and scale up of programs. Second, Avahan staff stated that they shared data on trends in the epidemic with counterparts at NACO as it became available, however frequently this data was shared in discussions within government prior to it having been formally written up, and was not presented in the form of scientific papers or summaries. Accordingly respondents within government may have been exposed to this evidence, but not necessarily viewed it as scientific data or research evidence, perhaps rather viewing it as expert advice on the nature of the unfolding epidemic.

In this case, a hallmark of the nature of communication, was that most critical communication occurred through the ‘inside track.’ Virtually all the actors interviewed, pointed to the fact that Avahan had effectively embedded itself within NACO, as being key to the influence that it wielded. The BMGF is in many respects a non-traditional donor. Unlike the World Bank or well-established bilateral development agencies, it does not have clearly defined channels for communicating with government (for example through systems of aide-memoires, missions, etc). Even within the BMGF, the Avahan program was atypical in terms of the intensity of its interaction with government. In terms of wielding influence, the position of the BMGF created both advantages and disadvantages. First, particularly during the early days of Avahan, the credibility of the program and hence evidence from the program was fragile, and Avahan did not have automatic access to policy discussions, compared to perhaps better-established development partners. However unlike some of the other partners, Avahan did have considerable flexibility in programming and this enabled it to be responsive and fast. Avahan’s ability to respond rapidly to NACO’s need for additional technical resources to support implementation enabled it to position itself to exert influence within NACO. It is possible, though not certain, that this strategy may have led to some resentment from other development partners, as described above.

BMGF brought significant financial resources to address the HIV/AIDS epidemic in India, and indeed 69% of the budget for NACP III is being met by development partners [22]. India, however, is obviously an emerging economic superpower and thus one might expect such financial contributions to secure less influence than in more donor-dependent contexts. Indeed, it appears that in order to exert policy influence, Avahan needed to deploy its support strategically, particularly through funding positions within NACO and the Technical Support Units.

### Limitations

This study set out to assess the impact of Avahan on the development of NACP III, and accordingly while probes in the interview guides did ask about the roles of other actors in influencing NACP III, this was not the focus of our analysis. The interviews were conducted several years after the first steps in development of NACP III policy, and thus it may have been difficult for respondents to remember precise details of discussions. While this may have been problematic, we do not believe that it led to any systematic bias in responses. There was also variation in the exposure of the respondents to the overall policy development process, which hindered the ability of some interviewees to respond to some of the questions in the discussion guide. There is also typically a tendency among respondents to portray their role in a positive manner. In this case, some of the respondents may have attributed a greater role for themselves and/or their organization in the development and implementation of NACP III. Similarly, there is an incentive for government respondents to minimize the influence of external agents in their retelling of policy development processes. While it is inevitable that some degree of bias is present in these results, the inclusion of multiple stakeholders in this analysis and triangulation between stakeholders helps to ensure a more comprehensive view of the process and mitigates to some extent, the impact of the potential bias.

## Conclusion

This study sought to assess the influence of one particular development partner on a specific policy in India, and the role that evidence played in mediating this relationship. While the BMGF has been a major funder of HIV/AIDS prevention in India, given the circumstances their financial clout was not the primary factor explaining any influence that they exerted. Instead Avahan’s ability to influence the policy debate rested primarily upon the knowledge and experience that the Avahan program embodied. While studies of knowledge translation typically focus primarily on scientific evidence, this study suggests that other forms of evidence, notably best practice evidence derived from program experience, and disseminated through personal communication, can be particularly influential. This best practice evidence was supplemented by the knowledge and experience of the many Foundation and Avahan partner staff who worked within government bringing tacit evidence from the Avahan experience to bear. Finally, and particularly during the policy formulation stage, field trips and personal testimony appear to have been effective influence strategies. This contrasts with typical studies of evidence influence that focus principally on the role of research.

The framework (Figure 2) that underpins this paper provides a useful tool to analyze how evidence is used to exert influence. To date, there have been relatively few attempts to document or analyze the instrumental use of evidence in policy debates, and we encourage the further application of this framework in studies of this nature. While the growing efforts to support knowledge translation in LMICs are valuable, they need to more clearly take account of the many ways in which evidence already enters policy debates, such as through the interventions of development partners.

## Abbreviations

AIDS: Acquired immuno deficiency syndrome; BMGF: Bill & melinda gates foundation; CBO: Community based organization; DFID: Department for international development; FSW: Female sex workers; GFATM: Global fund to fight aids, tuberculosis and malaria; HIV: Human immunodeficiency virus; M&E: Monitoring & evaluation; MIS: Monitoring & information system; MOHFW: Ministry of health and family welfare; MSM: Men who have sex with men; NACO: National AIDS control organization; NACP: National AIDS control program; NGO: Non-governmental organizations; NTSU: National technical support unit; SACS: State aids control society; STI: Sexually transmitted Infection; TI: Targeted interventions; TSG: Technical support group; TSU: Technical support unit; USAID: United States agency for international development.

## Competing interests

This research was funded by a grant from the Bill and Melinda Gates Foundation as part of a broader independent assessment of Avahan Phase 2. The views expressed herein are those of the authors and do not necessarily reflect the official policy or position of the Bill & Melinda Gates Foundation.

## Authors’ contributions

NT and SB developed the study design and survey tools with input from RB and SS. RB and SS conducted interviews. NT and SB led the data analysis and paper writing. RB and SS contributed to this process. All authors have read and approved the final manuscript.
